# Supplementary material for: Diagnostic accuracy of Savanna RVP4 (QuidelOrtho) for the detection of Influenza A virus, RSV, and SARS-CoV-2
Source: Microbiol Spectr. 2024 Jul 11;12(8):e01153-24. doi: 10.1128/spectrum.01153-24 (PMC11302293; doi:10.1128/spectrum.01153-24)
Supplement: Table S1 — Ct values for the calculation of the inter- and intra-assay precision. [file spectrum.01153-24-s0001.docx]

Table S1: Ct values for the calculation of the inter- and intra-assay precision

|  | Day 1 (Ct value) | | | Day 2 (Ct value) | | Day 3 (Ct value) | |
| --- | --- | --- | --- | --- | --- | --- | --- |
| Target | Test 1 | Test 2 | Test 3 | Test 1 | Test 2 | Test 1 | Test 2 |
| RSV | 20 | 20 | 20 | 19 | 19 | 19 | 19 |
| Flu A | 21 | 19 | 20 | 19 | 19 | 19 | 21 |
| SARS-CoV-2 | 17 | 18 | 17 | 17 | 17 | 17 | 17 |
